# Association of Single-Nucleotide Variants in ACE2 with the Persistence of Positive qPCR Test for SARS-CoV-2 in Healthcare Professionals During the First Wave of the COVID-19 Pandemic

**DOI:** 10.3390/microorganisms12122560

**Published:** 2024-12-12

**Authors:** Karina Jiménez-Gil, Jorge Alberto Cerón-Albarrán, Melissa Daniella Gonzalez-Fernandez, Rosalba Sevilla-Montoya, Alberto Hidalgo-Bravo, Javier Angeles-Martínez, Daniel Montes-Herrera, Oscar Villavicencio-Carrisoza, Carmen Selene García-Romero, José Esteban Muñoz-Medina, Irma Eloisa Monroy-Muñoz

**Affiliations:** 1 Reproductive and Perinatal Health Research Department, National Institute of Perinatology, Mexico City 11000, Mexico; 2 Genomics Medicine Department, National Institute of Rehabilitation, Mexico City 14610, Mexico; 3 Specialized Laboratories Division, Mexican Social Security Institute, Mexico City 06700, Mexico; 4 Immunobiochemistry Department, National Institute of Perinatology, Mexico City 11000, Mexico; 5 Infectology and Immunology Department, National Institute of Perinatology, Mexico City 11000, Mexico; 6 Quality of Supplies and Specialized Laboratories Coordination, Mexican Social Security Institute, Mexico City 37320, Mexico

**Keywords:** SARS-CoV-2 RT–qPCR, positivity, persistence, healthcare workers, ACE2 genetic variants, first wave

## Abstract

The persistence of qPCR positivity for SARS-CoV-2 in individuals who recovered from COVID-19 raised several questions regarding viral transmission, with a special interest in healthcare professionals who may pose a risk of transmitting SARS-CoV-2. This issue highlights the necessity for identifying the genetic risk factors associated with persistent SARS-CoV-2 infection. A promising target for achieving this goal is the angiotensin-converting enzyme 2 (*ACE2*) gene, which has been associated with clinical characteristics of COVID-19 infection, such as severity. The analysis of samples from the first wave of the COVID-19 pandemic represents the initial response of the immune human system against this new virus, without the effect of vaccination or the presence of multiple strains. The aim of this study was to analyze the association of genetic variants in *ACE2* with persistent SARS-CoV-2 infection. We conducted a case–control study, including 151 healthcare workers who tested positive for SARS-CoV-2 by qPCR during the first wave of the COVID-19 pandemic, and who were followed up until their results were negative. *ACE2* was sequenced through Sanger sequencing. The sequence was compared against a reference sequence and variants identified. Four *ACE2* variants were associated with persistent SARS-CoV-2 qPCR positivity. Three of the variants with an effect on the resulting protein were associated with increased risk of persistent SARS-CoV-2 qPCR positivity, NG_012575.2:g.35481 C>T, NG_012575.2:g.35483 G>T and NG_012575.2:g.35498 G>T. On the other hand, the rs2285666 (NG_012575.2:g.14934 G>A) was associated with a higher risk for persistent SARS-CoV-2 qPCR positivity in women and rs4646150 (NG_012575.2:g.25701 G>A) in men. The NG_012575.2:g.35498 G>T variant represents an amino acid change with a possibly harmful effect on ACE2 function. Our results suggest that *ACE2* variants might be useful for identifying the population at higher risk for developing persistent SARS-CoV-2-positive qPCR results. This knowledge can be helpful for designing health policies for protecting healthcare professionals and, in consequence, users of health services.

## 1. Introduction

Since its first appearance in 2019, SARS-CoV-2 has infected over 776 million and caused over 7 million deaths around the world [1].

The public health impact of the COVID-19 pandemic has resulted in a great effort by the scientific community to elucidate the underlying mechanisms of all of the spectrums of COVID-19. The disease spectrums range from asymptomatic to the severe forms of the disease, and of those that do not seem to follow the same evolution as long COVID.

As this disease is caused by coronavirus 2 (SARS-CoV-2) infection, much of the related research has focused on the mechanisms of SARS-CoV-2 entry into host cells and, particularly, on the binding of the viral spike (S) protein to its host receptor, angiotensin-converting enzyme 2 (ACE2) [2,3].

The *ACE2* gene is located on chromosome Xp22.2 and encodes a type 1 transmembrane protein. ACE2 has a peptidase domain (PD) and a collectrin-like domain (CLD). ACE2 has carboxypeptidase activity, which has been the main interest of most studies of ACE2 function, as this activity is responsible for the degradation of angiotensin II (Ang II), producing the peptide Ang1–7 [4].

Imbalances in ACE2 activity have been proposed to contribute to chronic diseases such as diabetes and hypertension, and consequently have been associated with a greater risk of developing severe COVID-19. Changes in ACE2 function following SARS-CoV-2 infection could affect more individuals with previous altered ACE2 expression caused by obesity, diabetes or hypertension [5], and it is very important to highlight that these comorbidities, together with old age, male sex, and racial/ethnic disparities have been associated with the development of severe COVID-19 [6].

In addition, the impact of genetic polymorphisms in *ACE2* plays an important role as an additional related host entry factor, which is why the impact of structural and regulatory genetic variants of *ACE2* on COVID-19 susceptibility is an important issue for further investigation [7,8]. African populations have shown a genetic predisposition to express significantly lower levels of ACE2, and this finding could explain the lower incidence of COVID-19 in Africans. In contrast, allele frequencies contributing to higher ACE2 expression are observed in South Asia [9,10].

Disease severity and infection have been widely studied, but there is another phenomenon known as positivity persistence of SARS-CoV-2 by RT–qPCR in individuals who have recovered from COVID-19 (from mild to severe forms of the disease), which has not been completely resolved. The duration of virus detection worldwide (using RT–qPCR) in asymptomatic or presymptomatic patients is highly variable. In a study that included 24 asymptomatic and presymptomatic patients, the average duration from the first positive test to the first of two consecutive negative tests was 9.5 days (with a time range of 1 to 21 days). The authors found that the virus was detected for a longer period in patients who subsequently developed symptoms (presymptomatic: n = 5, average 12 days) than in those who remained asymptomatic (n = 19, average 6 days) [11]. This persistence of positivity raises the question about the infectivity of the viral particles detected by RT–qPCR. To date, only a few studies have described the association of serial RT–qPCR tests with cell culture infection, and the results are highly variable [12,13,14].

Therefore, it is important to identify the population at greater genetic risk for developing persistent positivity, in order to develop adequate surveillance and confinement strategies for preventing further spreading of the infection, with particular focus on healthcare professionals who are continually exposed to the virus and represent a potential source of contagion [15,16]. In a meta-analysis of COVID-19 in healthcare workers, made by Gómez-Ochoa in 2021, the data of 97 studies were analyzed. Anosmia, fever, and myalgia were identified as the only symptoms associated with SARS-CoV-2 positivity. The pooled prevalence of comorbidities was evaluated among the 11,772 positive healthcare workers, showing that 7% (95% CI; 4–10%; *p*-value for heterogeneity: 0.35, I2: 10%) had hypertension, 3% (95% CI; 1–8%; *p*-value for heterogeneity: 0.19, I2: 39%) had cardiovascular disease, 4% (95% CI; 2–7%; *p*-value for heterogeneity: 0.01, I2: 63%) had type 2 diabetes, and chronic obstructive pulmonary disease was observed in 3% (95% CI; 1–6%; *p*-value for heterogeneity: 0.01, I2: 77%) [17]. The advances in vaccination and treatment have allowed the weekly cases to decrease by 41% and weekly deaths to decrease by 13%, as reported by the World Health Organization in the COVID-19 Epidemiological Update [18]. Although this panorama is encouraging, SARS-CoV-2 infection and COVID-19 still need further investigation.

## 2. Materials and Methods

### 2.1. Study Population

This was a cross-sectional exploratory study including 151 workers of the National Institute of Perinatology in Mexico City who tested positive for SARS-CoV-2 by RT–qPCR and who provided written informed consent. This study was conducted according to the guidelines of the Declaration of Helsinki and approved by the Ethics and Scientific Committees of the National Institute of Perinatology with the registration number 2020-1-27.

Convenience sampling of consecutive patients was performed. All workers who tested positive for SARS-CoV-2 by RT–qPCR and who were followed up until they tested negative during the period from March 2020 to December 2020 were included. The sample population was separated into two groups. The case group or persistent group included workers who tested positive for SARS-CoV-2 by RT–qPCR for more than 15 days (n = 67). The control group or non-persistent group included workers who tested positive for SARS-CoV-2 by RT–qPCR for fewer than 15 days (n = 75). Few demographic data were obtained, only data from sex, age, and presence of symptoms (comprising the most common ones, such as headache, fever, anosmia, etc.) were included (Table 1).

### 2.2. Genetic Variant Analysis

As described in previous studies made by our group, DNA extraction and high-resolution melting (HRM) followed by capillary sequencing were used to detect genetic variants of the *ACE2* gene [19]. A sample of peripheral blood was taken with a BD Vacutainer EDTA K2^®^ (Franklin Lakes, NJ, USA) blood collection system. Leukocyte DNA was isolated using the Promega Wizard^®^ Genomic DNA Purification Kit (Madison, WI, USA). The DNA samples were quantified with a Thermo Scientific NanoDrop™ 2000 instrument (Waltham, MA, USA), aliquoted and stored at −20 °C until use.

The high-resolution melting (HRM) method was employed to identify genetic variants in the ACE2 gene. Primers for HRM were designed using the Primer Select program (DNASTAR) (https://www.dnastar.com/software/, accessed on 5 April 2024), ensuring they did not form secondary structures during PCR, which could complicate the interpretation of melting profiles. Primer specificity was verified using the Primer Blast platform (NCBI) (https://www.ncbi.nlm.nih.gov/tools/primer-blast/, accessed on 5 April 2024). These primers targeted the complete exonic sequences along with small flanking intronic regions.

HRM reactions were carried out in a 20 µL mixture containing 5 µL of Milli-Q water, 1.5 µL of each primer at 20 pmol/µL, 10 µL of Bio-Rad Precision Melt Supermix (Hercules, CA, USA), and 2 µL of DNA at a concentration of 150 ng/µL. Amplification conditions included an initial denaturation step at 95 °C for 4 min, followed by 30 cycles of denaturation at 94 °C for 30 s, annealing for 30 s, extension at 72 °C for 30 s, and a final extension at 72 °C for 5 min. Melting curve analysis was conducted with parameters of 30 s at 95 °C and 30 s at 75 °C. Data collection occurred from 75 °C to 95 °C in 0.1 °C increments every 10 s using the CFX-96 Touch Real-Time PCR System (Bio-Rad, Hercules, CA, USA). Each HRM reaction was standardized, and all samples were analyzed in triplicate. Melting curve analysis was performed with Bio-Rad Precision Melt Analysis Software v.1.3 (Hercules, CA, USA). Samples displaying melting curve deviations from the average (the most frequently observed curve) underwent capillary sequencing to identify sequence variations responsible for the observed differences. Additionally, three randomly selected samples with typical melting curves were sequenced to confirm alignment with the consensus sequence from the NCBI RefSeq database (https://www.ncbi.nlm.nih.gov/refseq/, accessed on 5 April 2024).

### 2.3. DNA Sequencing

After purification with Thermo Fisher Scientific PCR ExoSAP-IT^TM^ (Waltham, MA, USA), HRM products were sequenced using Applied Biosystems Big Dye Terminator v1.1 and v3.1 kits (Applied Biosystems, Waltham, MA, USA) and an ABI PRISM 3130 DNA Analyzer. The obtained sequences were analyzed with BioEdit v.7.2 software and the NCBI Nucleotide Blast platform (Blastn) (https://blast.ncbi.nlm.nih.gov/Blast.cgi, accessed on 10 August 2022). Nucleotide sequences were translated using the translation tool in the ExPASy Bioinformatics Resource Portal (https://www.expasy.org/, accessed on 13 September 2022). Whenever a sequence variant was identified, the sample was sequenced again from the opposite direction to confirm the nucleotide change [19].

### 2.4. Bioinformatic Analysis

The dbSNP (https://www.ncbi.nlm.nih.gov/snp/, accessed on 20 November 2024) and ClinVar (https://www.ncbi.nlm.nih.gov/clinvar/, accessed on 20 November 2024) NCBI databases were searched for all identified variants. The variants were also analyzed with the online LUMC Mutalyzer 3 software (https://mutalyzer.nl/, accessed on 20 November 2024) to predict if the nucleotide change could be traduced into an amino acid change in the protein sequence.

The variants that alter the amino acid sequence were analyzed with the PolyPhen v2.0.23 bioinformatics tool (http://genetics.bwh.harvard.edu/pph2/, accessed on 20 November 2024), which was used to classify the variants as probably damaging, possibly damaging, or benign. The PolyPhen-2 tool assigns a score ranging from 0.0 to 1.0 to each variant: score values ≤0.446 are considered benign, score values between 0.446 and 0.908 are considered possibly pathogenic/damaging, and score values ≥ 0.908 are considered probably damaging.

For nonsynonymous SNPs, the Protein ANalysis THrough Evolutionary Relationships (PANTHER) Classification System (http://www.pantherdb.org/, accessed on 12 December 2022) and the Sorting Intolerant From Tolerant (SIFT) tool (http://sift-dna.org, accessed on 20 November 2024) were used. PANTHER estimates the probability that the SNP could affect the protein function, by calculating the period of time (in millions of years) during which a given amino acid has been conserved. SIFT analyzes data of sequence homology and amino acid physicochemical properties to predict if an amino acid change could affect protein function.

Synonymous variants were analyzed with the Human Splicing Finder (HSF) 3.0 tool (http://www.umd.be/HSF3/, accessed on 20 November 2024) to determine if the detected variants create alternative donor and acceptor sites, or modify branch point breaking, or create silencers and remove enhancers.

Reported genetic variants were analyzed in the Ensembl database with the Variant Effect Predictor (VEP) tool (https://grch37.ensembl.org/Homo_sapiens/Tools/VEP, accessed on 20 November 2024), which helps to predict the functional consequences of known variants.

### 2.5. Molecular Modeling

The SignalP 5.0 server (https://services.healthtech.dtu.dk/services/SignalP-5.0/, accessed on 20 November 2024) was used to delete the signal peptide from the N-terminal region of ACE2. The homology model of ACE2 was produced using the NCB BLASTP tool (https://blast.ncbi.nlm.nih.gov/, accessed on 20 November 2024) and the ExPASy SWISS-MODEL (protein structure homology modeling) server (https://swissmodel.expasy.org/, accessed on 20 November 2024) based on the percentage of identity and similarity with the entered sequence. 7DQA, which corresponds to the cryo-EM structure of the SARS-CoV-2 complex, was used as a template. Modeler software v.10.1 (Biopharmaceutical Sciences and Pharmaceutical Chemistry, and California Institute for Quantitative Biomedical Research, Mission Bay Byers Hall, University of California San Francisco) was used to construct the model with the lowest energy value. The model was visualized with the UCSF Chimera bioinformatics program v.1.15 (Office of Cyber Infrastructure and Computational Biology, National Institute of Allergy and Infectious Diseases, USA). MatchMaker and Match align, tools available in UCSF Chimera v.1.15, were used to evaluate the model and observe the atoms of interest. MatchMaker aligns structures based on sequence or structural similarities. Match align, focuses on aligning specific chains or models for detailed evaluations. A limit of 5.0 Å is applied. The root mean square deviation (RMSD) was calculated (1.93) through the Protein Database Summaries (PDBsum) server (https://www.ebi.ac.uk/thornton-srv/databases/pdbsum/, accessed on 20 November 2024). The RMSD value can be used to analyze the degree of divergence in aligned structures, as it measures the average deviation between equivalent atoms after aligning the structures. Low RMSD values (typically < 2 Å) indicate high structural similarity. High RMSD values reflect significant differences in the structures or alignments.

I-mutant (http://folding.biofold.org/i-mutant/i-mutant2.0.html, accessed on 20 November 2024) was utilized to predict alterations in protein stability resulting from point mutations based on either the protein’s structure or sequence, while CUPSAT was specifically employed to assess changes in protein stability (https://cupsat.brenda-enzymes.org/ accessed on 20 November 2024) and was used to predict changes in protein stability. The prediction model uses amino acid atom potentials and torsion angle distributions to evaluate the amino acid environment of the mutation site. A positive value suggests the mutation thermodynamically stabilizes the protein, while a negative value indicates destabilization. In addition, this bioinformatic tool predicts and detects the changes in free energy (∆∆G) that occur when mutations are present during the protein folding–unfolding process. A positive value of ∆∆G indicates that the mutation is thermodynamically stable; in contrast, negative values indicate destabilization, so the magnitude of ∆∆G is indicative of the extent of the mutation. I-mutant is a server that predicts changes in protein stability after a point mutation based on its structure or sequence. It calculates a DDG value that describes the free Gibbs energy difference between the unfolded state of the mutated protein and the wild-type protein (kcal/mol). A DDG value < 0 indicates decreased stability and a DDG value > 0 indicates increased stability.

### 2.6. Statistical Analysis

The frequencies of *ACE*2 variants in workers with and without persistent SARS-CoV-2 positivity by RT–qPCR were determined. The Hardy–Weinberg equilibrium was evaluated by the chi-square test. Analysis was performed with the SPSS version 20.0 statistical package (SPSS, Chicago, IL, USA). Quantitative variables are expressed as the means ± standard deviations, and qualitative variables are expressed as proportions or percentages. Data distribution of quantitative variables was tested through a Shapiro–Wilk test. Differences in continuous variables between two groups were assessed with Student’s *t* test. Nominal variables were analyzed using the chi-square test. Bonferroni correction was used for multiple testing due to the increased risk of Type I error (0.05 divided by 2; *p* < 0.025).

## 3. Results

### 3.1. Population Sample Characteristics

A population sample of 151 workers from the National Institute of Perinatology with a positive SARS-CoV-2 RT–qPCR test who were followed up until they were negative participated in the study. Participants were divided into two groups: 67 persistent workers (23 males and 44 females) and 75 non-persistent workers (29 males and 46 females) (Table 1).

No statistically significant differences were found regarding sex or age (*p* = 0.606 and *p* = 0.203, respectively) (Table 1). A significant difference was found with respect to presence of symptoms (*p* = 0.016).

### 3.2. Genetic Variant Detection

A total of 10 genetic variants were found in the population sample (Table 2): 3 missense variants (NG_012575.2:g.35498 G>T, NG_012575.2:g.42984 A>G, and NG_012575.2:g.43103 C>G) and 7 synonymous variants (NG_012575.2:g.14934 G>A, NG_012575.2:g.25701 G>A, NG_012575.2:g.35481 C>T, NG_012575.2:g.35483 G>T, NG_012575.2:g.36793 T>C, NG_012575.2:g.42916 T>A, and NG_012575.2:g.43073 G>A). From this last group of variants, four of them were found in an intronic region (NG_012575.2:g.14934 G>A, NG_012575.2:g.25701 G>A, NG_012575.2:g.36793 T>C, and NG_012575.2:g.42916 T>A) and three in an exonic region (NG_012575.2:g.35481 C>T, NG_012575.2:g.35483 G>T, and NG_012575.2:g.43073 G>A).

As the ACE2 gene is located in the X chromosome, the genotype frequency was only assessed in the female population (Table 2).

The logistic regression analysis of the 10 variants (resumed in Table 2) showed that the 3 variants lying on Exon 13 (NG_012575.2:g.35481 C>T, NG_012575.2:g.35483 G>T, and NG_012575.2:g.35483 G>T) increase the risk of having persistent RT-qPCR positive results. These three variants exhibit the same OR because their allelic and genotypic frequencies are the same (Table 3).

When the results were adjusted by the presence of symptoms, we found a higher risk for developing persistence when one of these three variants was present (Table 4).

The analysis by chi-square test of the allelic frequencies of the genetic variants found in the male population showed the association of NG_012575.2:g.25701 G>A, NG_012575.2:g.35481 C>T, NG_012575.2:g.35483 G>T, and NG_012575.2:g.35498 G>T (Table 5).

The logistic regression analysis showed that the variant in exon 8 (NG_012575.2:g.25701 G>A) and the three variants located in exon 13 (NG_012575.2:g.35481 C>T, NG_012575.2:g.35483 G>T, and NG_012575.2:g.35483 G>T) increased the risk of having persistent RT-qPCR positive results (Table 6).

Bioinformatic analysis of the associated variants revealed that two variants were previously reported as a single-nucleotide variant. Analysis with the Ensembl database showed that NG_012575.2:g.14934 G>A, also known as rs2285666, is a genetic variant located in a splicing donor sequence that falls between the third and sixth bases after the splice junction (5′ end of the intron). NG_012575.2:g.25701 G>A, or rs4646150, is located in intron 7, with no associated biological or clinical consequences according to the Ensembl database analysis (Table 7).

Three other variants, NG_012575.2:g.35481 C>T, NG_012575.2:g.35483 G>T, and NG_012575.2:g.35498 G>T, have not been previously reported. The Ensembl database analysis showed that these three variants are located in the coding region of exon 13, but NG_012575.2:g35481 C>T and NG_012575.2:g.35483 G>T are synonymous variants, and only NG_012575.2:g.35498 G>T is a missense variant that generates a change from a lysine residue to an asparagine residue at position 600 of the amino acid sequence (Table 7).

Further analysis of NG_012575.2:g.35481 C>T and NG_012575.2:g.35483 G>T with the Human Splicing Finder Tool revealed that these two variants modify the nucleotide sequence of auxiliary regions involved in splicing mechanisms, such as exonic splicing enhancers or silencers. The analysis of NG_012575.2:g.35498 G>T with the PolyPhen tool predicted this variant to be possibly damaging, with a score of 0.804 (sensitivity: 0.84; specificity: 0.93). The SIFT tool predicted that this variant would affect protein function. Analysis with Panther revealed that this residue is not highly conserved among species, likely resulting in a benign variant (Table 7).

### 3.3. Molecular Modeling

**Figure 1:**
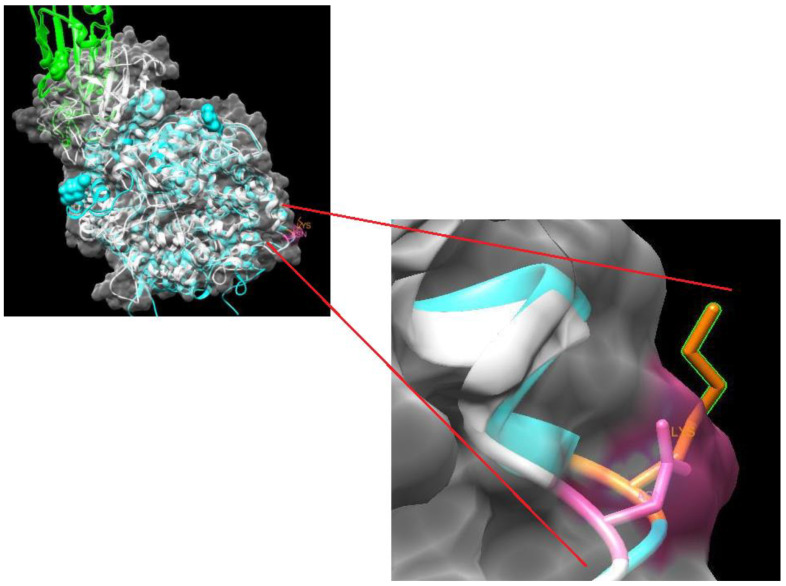
Homology model of ACE2. The structure of the employed template (7DQA) is shown in blue, and the Lys600Asn variant structure is shown in gray. The green region corresponds to the C chain of the spike glycoprotein of SARS-CoV-2. The lysine residue is highlighted in orange (Lys), and the asparagine residue (Asn) is highlighted in pink.

In the PDB, based on the obtained method, the resolution and the R values (smaller values were better), the best template was 7DQA. This model corresponds to the cryo-EM structure of the SARS-CoV-2 complex-RBD-ACE2. The modeler was used to build the model, and the model with the lowest energy value was selected. The model was visualized with the UCSF Chimera 1.15 bioinformatics program (Figure 1).

Tools such as MatchMaker and Match align were used to evaluate the model and observe the atoms of interest. The PDBsum server was used to verify whether the modeling was adequate through RMSD calculations. The RMSD value was 1.93, and 88.7% of the amino acids were located in the most favored positions.

For further analysis, other bioinformatics tools were employed, starting with the CUPSAT tool to predict changes in protein stability after point mutations. The K600N substitution is predicted to destabilize the overall stability of the protein, with an unfavorable torsion angle calculated from atomic potentials. The ∆∆G calculated for the variant was -0.98, indicating that the mutation is thermodynamically unstable. The results of I-mutant showed that the stability of the protein decreased in response to amino acid changes.

## 4. Discussion

The COVID-19 pandemic caught the entire world off guard, generating both economic losses and loss of human life. Healthcare personnel worldwide had to face this pandemic, accepting the impact that this could have on their own health as well as that of their families. The persistence of RT–qPCR positivity for SARS-CoV-2 in individuals who recovered from COVID-19 (from mild to severe forms of the disease) raises more questions about this new virus and the disease caused by it, with particular focus on healthcare professionals [16,17].

When age and sex variables were compared between the persistent and non-persistent groups, no statistically significant differences were found. However, a tendency toward a greater number of cases was observed in the female population in both study groups. This finding disagrees with worldwide reports, such as the one reported by Mattiuzzi and Lippi in 2020. They found a higher prevalence of infection in men than in women (71% versus 29%) among worldwide COVID-19 patients [18]. In Mexico, the data presented by Parra-Bracamonte in that same year showed a more balanced occurrence of cases but a greater frequency of male nonsurvivors (65.3%) [20]. Currently, data from the Health Secretary of Mexico, updated in August 2023, agree with the trend found in our study population, reporting that 53.7% of confirmed cases in our country are women and 46.3% are men [21]. Also, in a study carried out on the Italian population in 2021, where 377 patients were enrolled, female gender was associated with long COVID syndrome [22]. Although the persistence of RT–qPCR positivity for SARS-CoV-2 is not the same as long COVID syndrome, their behavior could be similar, given that a positive test leads to SARS-CoV-2 infection. Long COVID was primarily defined as experiencing at least 1 of the 25 symptoms listed by the World Health Organization for 30 or more days following a positive PCR test or, without a prior history of that symptom within 180 days before the SARS-CoV-2 infection. And persistent positivity refers to those cases in which patients continue to test positive in the RT-qPCR test for SARS-CoV-2 after their initial positive test, with or without the presence of symptoms [23,24,25].

Regarding symptoms, this term summarizes the occurrence of the most common ones, such as fever, headache, and anosmia. When the presence of them was compared between the persistent and non-persistent groups, a statistically significant difference was found, showing an association with the non-persistent group. This partially agrees with the findings of Fu in 2021. In this retrospective study, the authors described the patterns of viral polymerase chain reaction (PCR) positivity, finding an association of fever with a short viral positivity (within 21 days) [26]. Our study is limited by the fact that we do not evaluate individually the effect of each symptom. A meta-analysis conducted in 2021 found that 40% (95% CI: 17–65%) of the 230,398 healthcare workers with RT-qPCR-confirmed SARS-CoV-2 infection were asymptomatic at the time of diagnosis.

Of the 10 variants found (NG_012575.2:g.35498 G>T, NG_012575.2:g.42984 A>G, NG_012575.2:g.43103 C>G, NG_012575.2:g.14934 G>A, NG_012575.2:g.25701 G>A, NG_012575.2:g.35481 C>T, NG_012575.2:g.35483 G>T, NG_012575.2:g.36793 T>C, NG_012575.2:g.42916 T>A, and NG_012575.2:g.43073 G>A), 7 did not generate amino acid changes (Table 2). Previously, these types of mutations were considered biologically silent; however, it is now known that they can affect the function of the translated protein [27]. According to the literature, depending on the site of the noncoding region of the gene where the variants are found, different regulatory mechanisms may be modified and could alter cellular processes such as gene regulation; the structure of mRNA; and the processing, structure, and function of the resulting protein [28]. Genetic variants can be found in regulatory elements, such as conserved sequences of donor sites; branch points and the polypyrimidine tract; and intronic (ISE and ISS) and exonic (ESE and ESS) enhancers or silencers, which can change the primary structure of the protein since they are necessary to define intron–exon junctions in large eukaryotes, miRNAs, and binding sites for transcription factors, which can alter gene expression levels [29]. This is the case for the variants NG_012575.2:g.35481 C>T and NG_012575.2:g.35483 G>T, as they alter auxiliary sequences, such as the ESE/ESS motifs. Reports worldwide suggest that 5 to 10% of human genes contain at least one region where these types of mutations could be harmful [30].

NG_012575.2:g.14934 G>A, also known as rs2285666, has been widely described since the COVID-19 epidemic. It was reported as a splice donor region variant in the Ensembl database [31]. Alternative splicing is responsible for protein diversity in more than 90% of human genes [32,33], and genetic variants that cause defects in splicing have been proven to be the cause of multiple diseases [33,34], as the function and specificity of the resulting protein could change from the original form of the protein. The rs2285666 variant has also been classified as a target of an NMD transcript variant. According to the literature, this post-transcriptional mRNA quality control mechanism is responsible for the elimination of mRNAs that contain premature termination codons that could generate truncated proteins with predicted harmful effects on the organism. From a medical perspective, this finding suggests that the NMD pathway plays an important role in modulating the phenotypic outcome of genetic disorders [35]. Furthermore, according to the studies of diseases associated with the reported variants in the Ensembl database, rs2285666 has been related to non-insulin-dependent diabetes mellitus, hypertension, dyslipidemia, and orthostatic hypertension. Some studies have evaluated the association of rs2285666 with COVID-19 severity [32,33,34]. In the Iraqi population, in 2023, Adimulam reported an association between the rs2285666 T allele and an increased frequency of severe cases [36]. In contrast, in 2022, Alimoradi reported an association of GG genotype of rs2285666 with a greater risk of SARS-CoV-2 infection but not with COVID-19 severity [37]. In a study performed in a Latin American population (Colombian), no association was found between *ACE2* rs2285666 polymorphism and COVID-19 [38]. In our study population, the AA genotype was associated with a higher risk of SARS-CoV-2 persistent positivity of RT–qPCR in women. This finding appears consistent with those of other studies, as our analysis did not include severe cases and was not focused on evaluating SARS-CoV-2 infection itself; moreover, this phenomenon has not been completely elucidated, so we cannot confirm that the persistence of SARS-CoV-2 RT–qPCR positivity follows the same pathways as SARS-CoV-2 infection and COVID-19 severity.

NG_012575.2:g.25701 G>A, located in intron 7, was reported as rs4646150; however, there is no associated clinical information. According to Ensembl, it is a noncoding variant with no functional or structural consequences. Worldwide, the frequency of the wild-type G allele is 0.9952, while that of the variant allele is 0.0047. The same trend occurs in the Latin American population, with frequencies of C = 0.913 and A = 0.087, respectively. Although there is no functional, structural, or clinical evidence associated with this variant, in our study, the statistical association of this variant with a higher risk of persistence of SARS-CoV-2 RT–qPCR positivity in men highlights the necessity of further analysis of rs4646150.

The last variant, NG_012575.2:g.35498 G>T, is located in exon 13 and corresponds to the K600N amino acid change. PolyPhen analysis showed that it is a possibly harmful change, and the prediction is based on the multiple alignment of the protein residues and their conservation across different species; in this case, the amino acid in question is not highly conserved. SIFT, a tool that analyzes the amino acid in question according to its physicochemical properties, predicts that the substitution will not be tolerated with a score of 0.00, agreeing with the results of PolyPhen-2. The analysis of the physicochemical characteristics of the amino acid residues involved in the substitution revealed that lysine is a hydrophilic amino acid and chemically acts as a base since its side chain contains a proton-accepting amino group that often participates in the formation of hydrogen or ionic bonds. In contrast, asparagine is a polar uncharged amino acid. Also, the results obtained with CUPSAT server and I-mutant led us to propose that NG_012575.2:g.35498 G>T could modify the recognition of the viral spike (S) protein by ACE2.

It is relevant to highlight that variants that caused a significant protein effect, such as the alteration of regulatory regions (such as ESE/ESS motifs) by NG_012575.2:g.35481 C>T and NG_012575.2:g.35483 G>T and the change in amino acid sequence by NG_012575.2:g.35498 G>T, were associated with a greater risk of persistent SARS-CoV-2 RT–qPCR positivity in our study population. Therefore, a wider study of these variants should be conducted in other populations.

Our study has four important limitations. The first one relies on the potential impact of pre-existing comorbidities on increasing the morbidity of COVID-19 in adults. Although our study is not trying to associate the SARS-CoV-2 infection and COVID-19 development with ACE2 variants, it would have been relevant to evaluate how the presence of comorbidities could affect the association of ACE2 variants with the persistence of RT–qPCR positivity for SARS-CoV-2, but the lack of comorbidities data of the study population did not allow it. The second one is the lack of stratification of the symptoms present in our study population; the study of each symptom individually could have allowed a better understanding of the persistence phenomenon. The third one involves the timing of the study (March 2020 to December 2020). The present study was performed in the first phase of the pandemic before any vaccination scheme was available, and when only the Alpha variant of the virus was circulating in our country [39,40], so the effect of the vaccination and of the presence of more variants of SARS-CoV-2 on the persistence of RT–qPCR positivity could not be addressed. The last limitation is the lack of samples from healthy donors. In this regard, considering that the pandemic affected the majority of the Mexican population, we could not be sure of recruiting individuals without contact with SARS-CoV2. In addition, the allelic frequencies of the genetic variants were not related to the infection, and the aim of this study was to investigate if they were associated with the risk of persistence.

Finally, although the timing of the study represents one of the limitations of this study, it also represents one of the biggest strengths too, as the analysis of samples from the first wave of the COVID-19 pandemic represents the initial response of the immune human system against this new virus, without the effect of vaccination or the presence of multiple strains.

## 5. Conclusions

This is the first report of an association of NG_012575.2:g.35481 C>T, NG_012575.2:g.35483 G>T, and NG_012575.2:g.35498 G>T with a greater risk of persistent SARS-CoV-2 RT–qPCR positivity. The three variants are located in regions that have a tangible effect on the resulting protein.

Although the NG_012575.2:g.14934 G>A variant has been previously reported, it has even been associated with a greater risk of severe COVID-19. We identified the first association of this variant with a higher risk of persistent SARS-CoV-2 RT–qPCR positivity in women.

This is also the first association of NG_012575.2:g.25701 G>A with an increased risk of developing persistent SARS-CoV-2 RT–qPCR positivity in men.

Further studies of the persistence of SARS-CoV-2 RT–qPCR positivity are needed to better understand the phenomenon underlying this persistence and how and why it differs from SARS-CoV-2 infection and COVID-19 severity. The analysis of comorbidities, vaccination, and virus variants should be included. On the other hand, the analysis of samples from the first wave of the COVID-19 pandemic represents the initial response of the immunological human system against this new virus, without the effect of vaccination or the presence of multiple strains.

## Figures and Tables

**Table 1:** Characteristics of the study population.

	Non-Persistent	Persistent	*p*
Sex (F/M) *	46/29	44/23	0.606
Age (years) *	44 ± 12	42 ± 13	0.203
Symptoms ^+^	58.1	34.3	0.016

* The data are shown as the means ± SDs and frequencies. ^+^ Data presented as percentages. The term “symptoms” includes the most common ones, like headache, fever, and anosmia. F: Female. M: Male.

**Table 2:** Distribution and analysis of the frequency of genotypes of the variants present in the *ACE*2 gene in the female population.

	Genotype Frecuency (%)				
NG_012575.2:g.14934 G>A (rs2285666)	A/A	A/G	G/G	*p* *	*p* * HWE
Non-persistent (*n* = 46)	0.44	0.66	0.5	N.S.	0.0383
Persistent (*n* = 44)	0.56	0.34	0.5		
NG_012575.2:g.25701 G>A (rs4646150)	G/G	G/A	A/A		
Non-persistent (*n* = 46)	0.46	0.61	0	N.S.	3.8958
Persistent (*n* = 44)	0.54	0.39	0		
NG_012575.2:g.35481 C>T	C/C	C/T	T/T		
Non-persistent (*n* = 46)	0.62	0.21	0	0.001	2.1302
Persistent (*n* = 44)	0.38	0.79	0		
NG_012575.2:g.35483 G>T	G/G	G/T	T/T		
Non-persistent (*n* = 46)	0.62	0.21	0	0.001	2.132
Persistent (*n* = 44)	0.38	0.79	0		
NG_012575.2:g.35498 G>T	G/G	G/T	T/T		
Non-persistent (*n* = 46)	0.62	0.21	0	0.001	2.132
Persistent (*n* = 44)	0.38	0.79	0		
NG_012575.2:g.36793 T>C	T/T	T/C	C/C		
Non-persistent (*n* = 46)	0.52	0	0	N.S.	0.0028
Persistent (*n* = 44)	0.48	1	0		
NG_012575.2:g.42916 T>A	T/T	T/A	A/A		
Non-persistent (*n* = 46)	0.51	0.50	0	N.S.	1.74
Persistent (*n* = 44)	0.49	0.50	0		
NG_012575.2:g.42984 A>G	A/A	A/G	G/G		
Non-persistent (*n* = 46)	0.52	0	0	N.S.	0.0028
Persistent (*n* = 44)	0.48	1	0		
NG_012575.2:g.43073 G>A	G/G	G/A	A/A		
Non-persistent (*n* = 46)	0.5	0.75	0	N.S.	0.046
Persistent (*n* = 44)	0.5	0.25	0		
NG_012575.2:g.43103 C>G	C/C	C/G	G/G		
Non-persistent (*n* = 46)	0.55	0.42	0	N.S.	2.1302
Persistent (*n* = 44)	0.45	0.58	0		

HWE: Hardy–Weinberg equilibrium. Comparisons were made using * chi-square test. N.S., non-significant.

**Table 3:** Logistic regression analysis of allelic and genotypic frequencies of female population. Non-adjusted model.

	*p*	OR	95% C.I.	
NG_012575.2:g.35481 C>T	0.001	6.232	2.067	18.789
NG_012575.2:g.35483 G>T	0.001	6.232	2.067	18.789
NG_012575.2:g.35498 G>T	0.001	6.232	2.067	18.789

OR: Odds Ratio.

**Table 4:** Logistic regression analysis of allelic and genotypic frequencies of female population. Adjusted models by symptoms.

	*p*	OR	95% C.I.	
NG_012575.2:g.14934 G>A	0.031	3.796	1.127	12.778
NG_012575.2:g.35481 C>T	0.000	19.275	4.480	82.930
NG_012575.2:g.35483 G>T	0.001	19.275	4.480	82.930
NG_012575.2:g.35498 G>T	0.001	19.275	4.480	82.930

OR: Odds Ratio.

**Table 5:** Distribution and analysis of the allelic frequency of the variants present in the *ACE*2 gene in the male population.

	Allelic Frecuency (%)		
NG_012575.2:g.14934 G>A (rs2285666)	G	A	p *
Non-persistent (*n* = 29)	0.75	0.47	N.S.
Persistent (*n* = 23)	0.25	0.53	
NG_012575.2:g.25701 G>A (rs4646150)	G	A	
Non-persistent (*n* = 29)	0.42	0.79	0.011
Persistent (*n* = 23)	0.58	0.21	
NG_012575.2:g.35481 C>T	C	T	
Non-persistent (*n* = 29)	0.74	0.22	<0.0001
Persistent (*n* = 23)	0.26	0.78	
NG_012575.2:g.35483 G>T	G	T	
Non-persistent (*n* = 29)	0.74	0.22	<0.0001
Persistent (*n* = 23)	0.26	0.78	
NG_012575.2:g.35498 G>T	G	T	
Non-persistent (*n* = 29)	0.62	0.21	0.001
Persistent (*n* = 23)	0.38	0.79	
NG_012575.2:g.36793 T>C	T	C	
Non-persistent (*n* = 29)	0.56	0	N.S.
Persistent (*n* = 23)	0.44	0	
NG_012575.2:g.42916 T>A	T	A	
Non-persistent (*n* = 29)	0.56	0.57	N.S.
Persistent (*n* = 23)	0.44	0.43	
NG_012575.2:g.42984 A>G	A	G	
Non-persistent (*n* = 29)	0.55	0.63	N.S.
Persistent (*n* = 23)	0.45	0.38	
NG_012575.2:g.43073 G>A	G	A	
Non-persistent (*n* = 29)	0.56	0	N.S.
Persistent (*n* = 23)	0.44	0	
NG_012575.2:g.43103 C>G	C	G	
Non-persistent (*n* = 29)	0.56	0	N.S.
Persistent (*n* = 23)	0.44	0	

* chi-square test. N.S., non-significant.

**Table 6:** Logistic regression analysis of allelic frequencies in male population.

	*p*	OR	95% C.I.	
NG_012575.2:g.25701 G>A	0.014	5.089	1.385	18.696
NG_012575.2:g.35481 C>T	0.001	9.722	2.527	37.402
NG_012575.2:g.35483 G>T	0.001	9.722	2.527	37.402
NG_012575.2:g.35498 G>T	0.002	8.125	2.129	31.007

**Table 7:** In silico analysis of associated variants.

Variant	Ensembl Database Analysis	Further In Silico Analysis
NG_012575.2:g.14934 G>A (rs2285666)	Splice donor region variant (47%) Intron variant (47%) NMD transcript variant (7%)	ClinVar: Not reported
NG_012575.2:g.25701 G>A (rs4646150)	Intronic variant	ClinVar: Not reported.
NG_012575.2:g.35481 C>T c.1783 C>T	Exonic variant Synonymous variant	HSF: Disturb auxiliary sequences. Significant disturbance of ESE/ESS motif proportion
NG_012575.2:g.35483 G>T c.1785 G>T	Exonic variant Synonymous variant	HSF: Disturb auxiliary sequences. Significant disturbance of ESE/ESS motif proportion
NG_012575.2:g.35498 G>T	Missense variant. Substitution of lysine for asparagine in position 600 of the protein sequence.	PolyPhen: Possibly damaging. Panther: Most likely, benign SIFT: The substitution is predicted to affect protein function.

HSF: Human Splicing Finder; ESE: exonic splicing enhancer; ESS: exonic splicing silencer; SIFT: Sorting Intolerant from Tolerant.

## Data Availability

The original contributions presented in this study are included in the article. Further inquiries can be directed to the corresponding author.
